# miR-17-5p and miR-4443 Promote Esophageal Squamous Cell Carcinoma Development by Targeting TIMP2

**DOI:** 10.3389/fonc.2021.605894

**Published:** 2021-10-27

**Authors:** Xiaojun Wang, Jiayi Han, Yatian Liu, Jingwen Hu, Ming Li, Xi Chen, Lin Xu

**Affiliations:** ^1^ Department of Thoracic Surgery, Nanjing Medical University Affiliated Cancer Hospital and Jiangsu Cancer Hospital and Jiangsu Institute of Cancer Research, Cancer Institute of Jiangsu Province, Nanjing, China; ^2^ Tianjin Medical University Cancer Institute and Hospital, National Clinical Research Center for Cancer, Tianjin’s Clinical Research Center for Cancer, Key Laboratory of Cancer Prevention and Therapy, Tianjin Medical University, Tianjin, China; ^3^ Department of Radiotherapy, The Affiliated Cancer Hospital of Nanjing Medical University & Jiangsu Cancer Hospital & Jiangsu Institute of Cancer Research, Nanjing, China; ^4^ State Key Laboratory of Pharmaceutical Biotechnology, Collaborative Innovation Center of Chemistry for Life Sciences, Jiangsu Engineering Research Center for MicroRNA Biology and Biotechnology, NJU Advanced Institute for Life Sciences (NAILS), School of Life Sciences, Nanjing University, Nanjing, Jiangsu, China

**Keywords:** miR-17-5p, miR-4443, ESCC, TIMP2, cancer development

## Abstract

**Background:**

Esophageal squamous cell carcinoma (ESCC) is one of the most frequently diagnosed cancers in the world with a high mortality rate. The mechanism about ESCC development and whether miRNAs play a critical role remains unclear and needs carefully elucidated.

**Materials and Methods:**

High-throughput miRNA sequencing was used to identify the different expression miRNAs between the ESCC tissues and paired adjacent normal tissues. Next, both CCK-8, Transwell and apotosis assay were used to evaluate the role of miRNA in ESCCcells. In addition, we used bioinformatic tools to predict the potential target of the miRNAs and verified by Western Blot. The function of miRNA-target network was further identified in xenograft mice model.

**Results:**

In ESCC, we identified two miRNAs, miR-17-5p and miR-4443, were significantly upregulated in ESCC tissues than adjacent normal tissues. TIMP2 was proved to be the direct target of both two miRNAs. The miR-17-5p/4443- TIMP2 axis was shown to promote the tumor progression *in vitro* and *in vivo* experiments.

**Conclusions:**

This study highlights two oncomiRs, miR-17-5p and miR-4443, and its potential role in ESCC progression by regulating TIMP2 expression, suggesting miR-17-5p and miR-4443 may serve as a novel molecular target for ESCC treatment.

## Introduction

Esophageal cancer (EC), one of the most frequently diagnosed cancers in the world, has the highest incidence rate in Eastern Asia (1). It has two main components: esophageal squamous cell carcinoma (ESCC, for about 80% of all ECs), and esophageal adenocarcinoma (for about 20% of all ECs). Due to the development of diagnosis and treatment techniques, the prognosis of ECs has been improved in the past decades. But it’s still far from satisfactory for its high recurrence rate and poor 5-year survival rate. Research indicates that over 80% of EC patients were dead within 5 years (2). Thus, exploring the key factors that cause the occurrence and development of EC may give evidence to the clinical treatment and improve the prognosis of EC.

MicroRNAs (miRNAs), which consisting of 18-22 nucleotide base pairs, has been verified to play important roles in regulating gene expression (3). The abnormal expression of miRNAs has been found in almost all kinds of tumors including EC (4). miRNA can play an oncogenic role or a tumor-suppressive role to affect the proliferation, migration or apoptosis of tumor cells. Among them, overexpression of miR-17-5p has been reported in various human cancers, including breast cancer (5), prostate cancer (6), hepatocellular carcinoma (7), pancreatic cancer (8), gastric cancer (9) and so on. Besides, miR-4443 was also shown to be upregulated in lung cancer (10) and breast cancer (11). It’s has also been reported that miR-17-5p can directly target ETV resulting in suppressing cell proliferation and invasion in triple-negative breast cancer (12), while it can also enhance cell proliferation in pancreatic cancer by targeting RBL2/E2F4 (13). However, the precise role of miR-17-5p and miR-4443 in EC has not been fully understood.

In this study, we identified the overexpression of miR-17-5p and miR-4443 in EC tissues compared to their paired adjacent tissues. Overexpression of miR-17-5p and miR-4443 promote EC cells’ proliferation and migration as well as reduces the expression of TIMP2, while down-regulation of miR-17-5p and miR-4443 got the opposite effect. We testified the important roles of miR-17-5p and miR-4443 in the development of EC and these results may provide new strategies for ESCC treatment.

## Material and Methods

### Tissues Specimens and Cell Lines

20 ESCC tissue samples and their paired adjacent paratumor normal tissues were collected from Jiangsu Cancer Hospital (Nanjing, Jiangsu, China). All patients signed the informed consents and the Ethics Committee of the Jiangsu Cancer Hospital approves the whole study. The protocol used in this study was based on approved guidelines by Ethics Committee of the Jiangsu Cancer Hospital. All patients were diagnosed ESCC by histopathology examination, and none of them had diagnosed other malignant tumor or received neoadjuvant chemotherapy or radiotherapy. All samples were immediately cut into small pieces after surgical resection and keep in liquid nitrogen until use.

The human esophageal cancer cell line, TE-10, and ECA-109 were purchased from Cell Bank of Chinese Academy of Science, Shanghai, China. All cells were cultured in RPMI-1640 (Gibco Life Technologies, Waltham, MA USA) containing 10% fetal bovine serum (FBS), 100 units/mL penicillin, and 100ug/mL streptomycin. The humidified incubator was set at 37°C containing 5% CO2.

### High-Throughput miRNA Sequencing

Total RNA extracted from 3 ESCC tissues and its paired adjacent normal tissues were used for high-throughput miRNA sequencing. The detailed procedure was the same as previously described (14).

### RNA Extraction and Real-Time qRT-PCR

Total RNA was extracted from cells or tissues by TRIzol reagent (Invitrogen) and quantified by NanoDrop spectrophotometer. TaqMan miRNA probes (Applied Biosystems, Foster City, CA) were used to quantify the miRNAs. All procedures were performed as previously described (15). And all of the experiments were run in triplicate. The miRNA internal control was U6 small nuclear RNA. After the completion of the reactions, the 2^-△△CT^ method was used to compare the relative quantification of each miRNA between every group.

### Cell Transfection

All of the miRNA mimics, inhibitors and scrambled negative control used in this research were designed and synthesized by GenePharma (Shanghai, China). The sequence of mature miR-17-5p and miR-4443 are 5’-CAAAGUGCUUACAGUGCAGGUAG-3’ and 5’-UUGGAGGCGUGGGUUUU-3’, respectively. Lipofectamine 2000 (Invitrogen, USA) was used as the cell transfection reagent and performed according to the manufacturer’s instructions.

### Cell Proliferation Assay

TE-10 and ECA-109 cells were seeded in the 6-well plate. 6h after transfection, the cells were reseeded to a 96-well plate at a density of 5×10^3^ cells per well. A Cell Counting Kit-8 assay (CCK-8) was performed at 0, 24h, 48h, and 72h respectively. The absorbance of the 450nm laser was measured after 2-hour incubation of cells and CCK-8. Each group had at least 5 repeats and all experiments were performed in triplicate.

### Cell Migration and Apoptosis Assay

Transwell assay was used to test the cells migration ability. In brief, the transwell chamber with 8μm pore polycarbonate membranes was put into a 24-well plate. A total number of 1×10^5^ cells suspended with serum-free RPMI-1640 were added into the upper chamber and 500μl RPMI-1640 with 10% FBS was added to the lower chamber. After 16h incubation, the cells in the upper chamber was wiped with a cotton swab and the cells migrated to the lower surface were fixed with 4% paraformaldehyde and stained with 0.05% crystal violet. The stained cells were then quantified by a spectrophotometer at 3 random areas. The apoptosis of cancer cells was tested by Annexin V-FITC/PI staining kit (BD Biosciences, San Diego, CA, USA). Besides, the total apoptotic cells were counted as the sum of early apoptotic (PI− AV+) and late apoptotic (PI+ AV+) cells.

### Luciferase Reporter Assay

The 3′-UTR of human TIMP2 containing putative binding sites was cloned into the p-MIR-REPORT plasmid (Ambion), and efficient insertion was confirmed by sequencing. To test the binding specificity, the sequences in human TIMP2 3′-UTR that interact with miRNA seed sequence were mutated. 293T cells were co-transfected with β-galactosidase (β-gal) expression plasmid (Ambion), a firefly luciferase reporter plasmid, and miRNA mimics or negative control. The β-gal plasmid was used as a transfection control. Luciferase activity was measured 24 h after transfection using a luciferase assay kit (Promega, Madison, WI, USA).

### Western Blot Analysis

The expression of TIMP2, as well as internal control GAPDH in cells and tissues, was assessed by western blot analysis. Homogenate tissues and cultured cells were lysed in RIPA buffer containing protease inhibitor cocktail. We used the 10% SDS-PAGE gels to separate the protein lysates, which was then electrically transferred to a polyvinylidene difluoride (PVDF) membranes. The membranes were then blocked by 5% skimmed milk for at least 1h at room temperature and followed on incubating with primary antibody (anti-TIMP2, 1:2000, Abcam, and anti-GAPDH, 1:3000, Abcam). After incubating with their specific second antibody at room temperature for 1h, the membranes were then visualized by ECL (Thermo Scientific, Rockford, USA) detection assay.

### Tumor Xenografts in Mice

All animals used in this study were approved by the ethics committee of Jiangsu Cancer Hospital and complied with NIH Guidelines. TE-10 cells were treated with miR-17-5p/miR-4443 overexpressing lentivirus or control lentivirus and were then injected subcutaneously into the inguinal folds of the nude mice at the concentration of 10^6^ cells per 0.2ml PBS. 28 days later, the mice were sacrificed and removed the xenografted tumors. The tumors were then measured the volumes and weights and then extracted protein for the TIMP2 expression detection.

### Statistical Analysis

All western blot images are representative of at least three independent experiments. Quantitative RT-PCR, luciferase reporter assay, cell proliferation, migration assay and cell apoptosis assay were performed in triplicate, and each experiment was repeated several times. Statistical analysis was calculated by SPSS 16.0. Presented data was carried out by at least 3 separate experiments and showed as mean ± SD. P<0.05 was considered statistically significant in this study by using the student’s t-test. *P<0.05, **P<0.01, ***P<0.001.

## Results

### High Expression of miR-17-5p and miR-4443 Was Observed in ESCC Tissue

**Figure 1:**
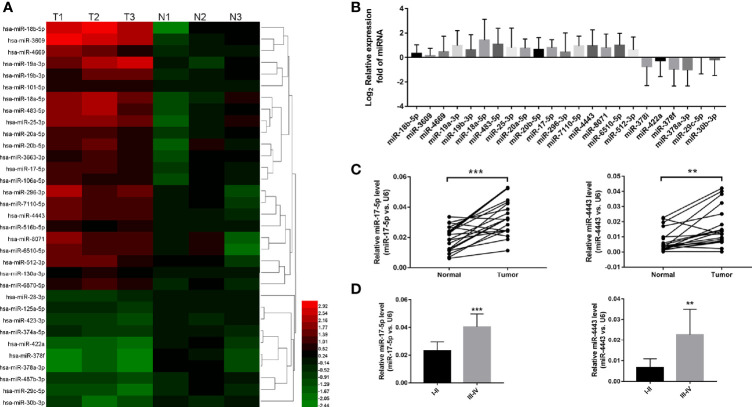
The expression level of miR-17-5p and miR-4443 in ESCC tissues. **(A)** high-throughput miRNA sequencing results. 23 miRNAs were statistically dysregulated, P<0.05, fold change>2 or <0.5. **(B)** Q-PCR analysis of all 23 screened miRNAs in 20 ESCC tissues and normal adjacent tissues. **(C)** Q-PCR analysis of the relative expression levels of miR-17-5p and miR-4443 in 20 pairs tissues. **(D)** The two miRNAs concentrations in different TNM stages (I–IV) of all 20 ESCC tissues. **P < 0.01, ***P < 0.001.

To explore the significantly expressed miRNAs in ESCC, we first use the high-throughput miRNA sequencing to identify the expression profiles of all miRNAs in the ESCC tissues and paired adjacent normal tissues. As shown in **Figure 1A**, among total 1295 miRNAs, 23 miRNAs were shown to be significantly dysregulated (P<0.05 and fold change > 2 or <0.5; 18 miRNAs were up-regulated and 5 miRNAs were down-regulated). We further validated all these 23 miRNAs levels by qRT-PCR in 13 ESCC tissues and their paired adjacent normal tissues (**Figure 1B**). And we found that two miRNAs (miR-17-5p and miR-4443) were stably up-regulated in ESCC (**Figure 1C**). So, the two miRNAs were selected as candicates for further investigation. Then we investigated the association between the two miRNAs expression and various clinicopathological variables in all samples. High correlation between miRNAs and tumor TNM stages was shown in **Figure 1D**, indicating that the two miRNAs signature is closely associated with ESCC progression.

### miR-17-5p and miR-4443 Promote Proliferation and Migration, and Inhibit Apoptosis *In Vitro*

**Figure 2:**
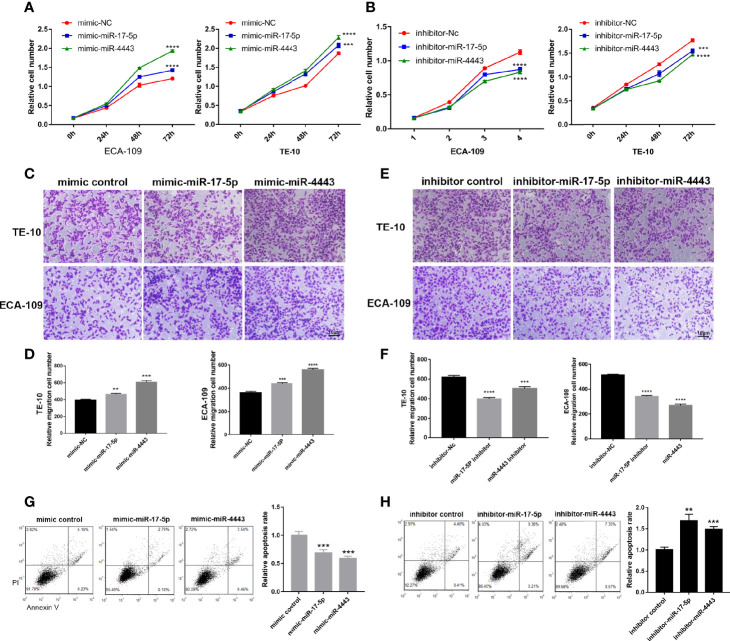
Effect of miR-17-5p and miR-4443 in the regulation of proliferation and migration of ESCC cells. **(A, B)** CCK8 assays were performed at 0h, 24h, 48h and 72h after the transfection of the ECA-109 cells and TE-10 cells with mimic-NC, mimic-miR-17-5p, mimic-miR-4443, inhibitor-NC, inhibitor-miR-17-5p and inhibitor-miR-4443. **(C, D)** Transwell analysis of the migration rate of ECA-109 and TE-10 cells transfected with an equal dose of mimic-NC, mimic-miR-17-5p, mimic-miR-4443. **(C)**, representative image; **(D)**, quantitative analysis. **(E, F)** Transwell analysis of the migration rate of ECA-109 and TE-10 cells transfected with equal dose of inhibitor-NC, inhibitor-miR-17-5p and inhibitor-miR-4443. **(E)**, representative image; **(F)**, quantitative analysis. **(G, H)** Analysis of apoptosis in TE-10 cells treated with mimic control, mimic-miR-17-5p, mimic-miR-4443, inhibitor control, inhibitor-miR-17-5p and inhibitor-miR-4443. The total apoptotic cells were counted as the sum of early apoptotic (PI− AV+) and late apoptotic (PI+ AV+) cells (left: representative image; right: quantitative analysis). **P < 0.01, ***P < 0.001, ****P < 0.0001.

To further explore the specific role of miR-17-5p and miR-4443 in the ESCC, we transfected the TE-10 and ECA-109 cells with miRNA mimics, inhibitors, and negative control then checked their effects on tumor behavior. As shown in **Figure 2A**, miR-17-5p or miR-4443 overexpression significantly promoted cell proliferation in both TE-10 and ECA-109, while downregulation showed the opposite effect (**Figure 2B**). In addition, transwell assay showed miR-17-5p and miR-4443 promoted cell migration ability in both cells (**Figures 2C, D**). Also, downregulation of miR-17-5p and miR-4443 reduced cell migration ability (**Figures 2E, F**). In the cell apoptosis assay, the percentage of apoptotic cells was significantly lower in TE-10 cells transfected with miR-17-5p or miR-4443 mimic (**Figure 2G**) and higher in cells transfected with miR-17-5p or miR-4443 inhibitor (**Figure 2H**). Taken together, these results suggest that miR-17-5p and miR-4443 may act as oncomiRs to promote ESCC progression.

### TIMP2 Is Identified as a Direct Target Gene to Both miR-17-5p and miR-4443

**Figure 3:**
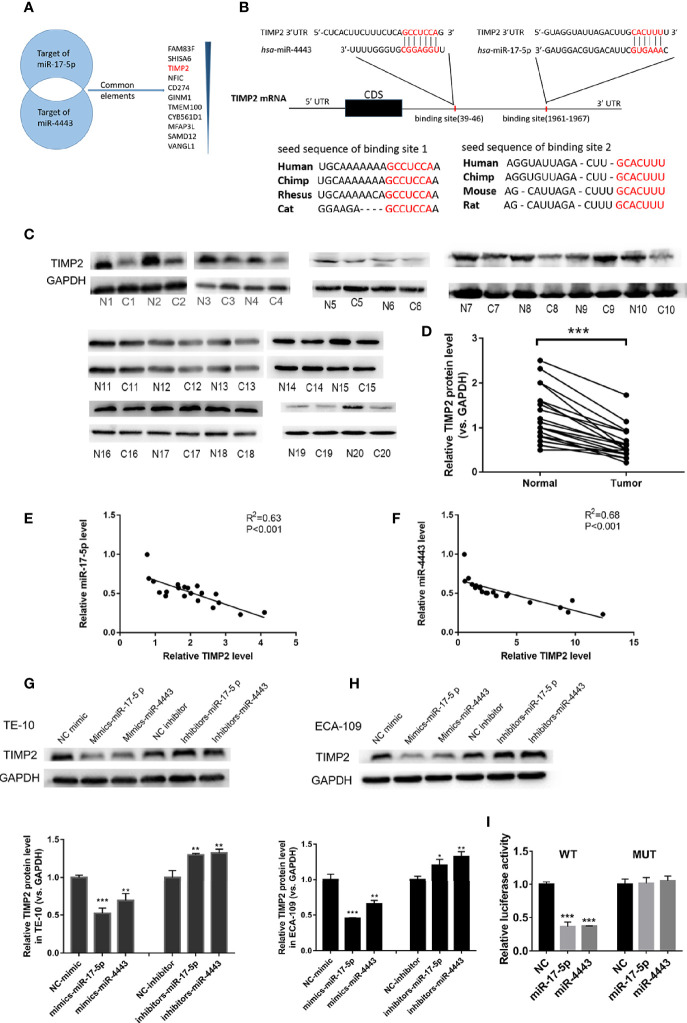
TIMP2 is the target of miR-17-5p and miR-4443 in ESCC cells. **(A)** The common targets of miR-4443 and miR-17-5p. All targets are predicted by two bioinformatics tools and the common targets are arranged by the confidence level. **(B)** Graphic description of the base-pairing interaction between miR-17-5p, miR-4443 and TIMP2 3’UTR and their exact position in the TIMP2 mRNA. **(C)** Western blot analysis of TIMP2 in 20 pairs of ESCC tissues. (N=Normal, C=Cancer). **(D)** Quantification of TIMP2 levels in ESCC tissues. **(E, F)** Pearson’s correlation scatter plot of the fold changes of miR-17-5p, miR-4443 and TIMP2 protein ESCC tissues. **(G, H)** The effect of transfection of inhibitor-miR or mimic-miR or their negative control on the expression of TIMP2 in two cell lines, TE-10 **(G)** and ECA-109 **(H)**. **(I)** Dual luciferase activity assay was used to detect the binding affinity between miR-17-5p, miR-4443 and TIMP2. All results were shown as mean ± SD (n = 3). *p < 0.05, **P < 0.01, ***P < 0.001.

To identify the direct target genes of miR-17-5p and miR-4443 in ESCC, we used two bioinformatics tools (TargetScan http://www.targetscan.org/vert_72/ and miRDB http://mirdb.org/). Because of miR-17-5p and miR-4443 having a similar effect on ESCC, we hypothesize if they could target the same protein. As shown in **Figure 3A**, TIMP2, the inhibitor of matrix metalloproteinases (MMPs), was considered to be a potential target with a high confidence level among all predicted common targets of miR-17-5p and miR-4443. The predicted site and their interaction between miR-17-5p, miR-4443 and 3’-UTR of TIMP2 was shown in **Figure 3B**.

miRNAs are known to play their role by inhibiting their target protein. We first investigated whether TIMP2 was down-regulated in ESCC tissues than paired adjacent normal tissues. As shown in **Figures 3C, D**, TIMP2 protein levels was significantly downregulated in ESCC tissues. To further clarify the relationship between miR-17-5p, miR-4443 and TIMP2, we performed a correlation analysis between miR- miR-17-5p, miR-4443 and TIMP2. According to the results, both the expression levels of miR-17-5p, miR-4443 are significantly and negatively correlated with TIMP2 protein level (**Figures 3E, F**). Moreover,in TE-10 cells, transfection of mimics-miR-17-5p or mimics-miR-4443-5p significantly inhibit TIMP2 expression, while downregulation of miR-17-5p or miR-4443 expression showed increased expression of TIMP2 (**Figure 3G**). These results were further verified in ECA-109 cells (**Figure 3H**).

To further confirm whether miR-17-5p and miR-4443 could directly target the predicted binding sites in the 3’-UTR of TIMP2, we performed luciferase reporter assays. The presumed binding sites of TIMP2 3’-UTR was designed to be inserted into a reporter plasmid which has a downstream firefly luciferase gene. We next transfected this recombined plasmid into 293T cells together with miRNA mimics or antisenses. As expected, transfection of mimics-miR-17-5p and mimics-miR-4443 significantly reduced the luciferase activity in A549 cells, while transfection their antisenses induced an increase in reporter activity (**Figure 3I**). Furthermore, we mutated the predicted binding sites in TIMP2 3’-UTR of both miRNAs and the luciferase activity resulting in not changing after either miRNAs overexpression. Thus, the results indicated that TIMP2 mRNA was the direct target of miR-17-5p and miR-4443.

### TIMP2 Attenuates the Effects of the miR-17-5p and miR-4443 in ESCC Cells

**Figure 4:**
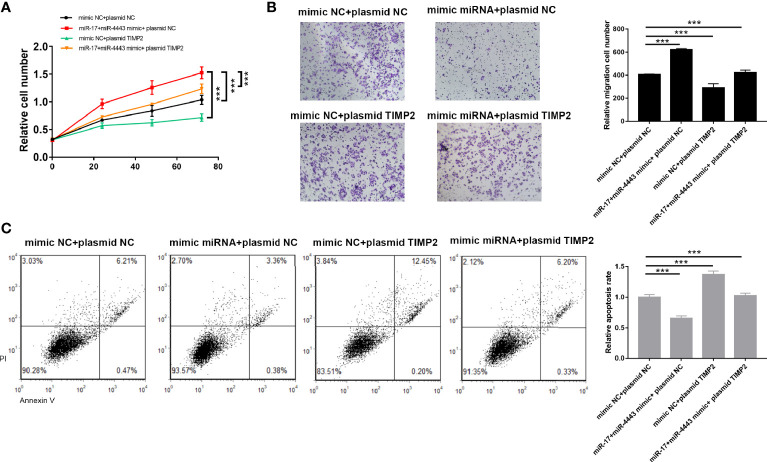
TIMP2 attenuates the effects of the miR-17-5p and miR-4443 in ESCC cells. **(A)** Analysis of proliferation **(A)**, migration **(B)** and apoptosis **(C)** in TE-10 cells treated with mimic-control plus plasmid control, mimic-miR-17-5p and mimic-miR-4443 mixture plus control vector, mimic-control plus TIMP2 plasmid, or mimic-miR-17-5p and mimic-miR-4443 mixture plus HIC1 TIMP2 plasmid. All results were shown as mean ± SD (n = 3). ***P < 0.001.

To test whether miR-17-5p and miR-4443 may suppress TIMP2 expression to affect cell proliferation, apoptosis and invasion, we transfected TE-10 cells with both mixture of mimic-miR-17-5p and mimic-miR-4443 and a plasmid designed to specially express the full-length ORF of TIMP2 without the miR-17-5p and miR-4443–responsive 3′-UTR. Proliferation, apoptosis and invasion assays revealed that ectopic expression of TIMP2 dramatically attenuated the inhibitory effect of the miR-17-5p and miR-4443 on cell apoptosis, and stimulatory effect on cell proliferation an invasion (**Figures 4A–C**).

### miR-17-5p and miR-4443 Promote ESCC Progression *In Vivo*

**Figure 5:**
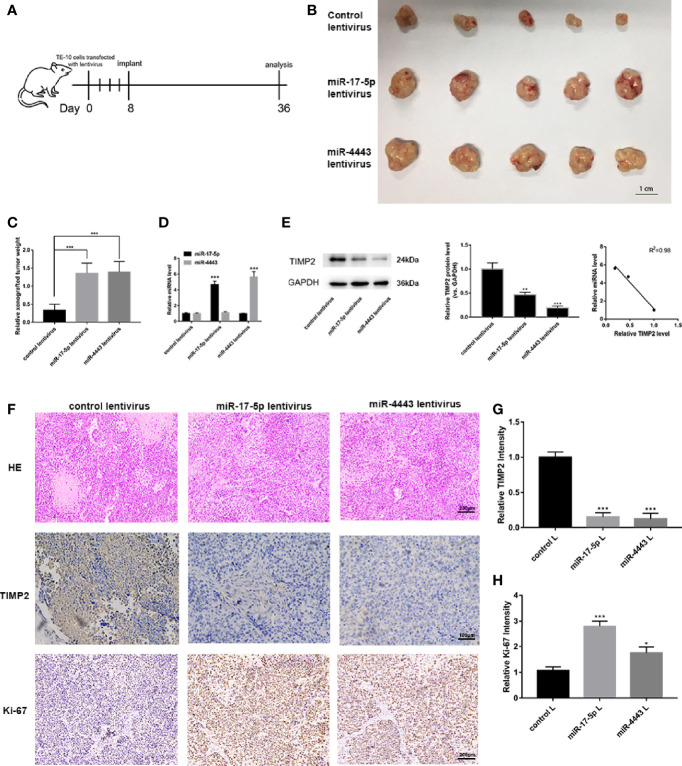
*In vivo* experiments to verify the effects of miR-17-5p and miR-4443 on ESCC cells. **(A)** The flow chart of the whole *in vivo* experiments. **(B)** The morphology of the isolated tumors in each group (n = 5). **(C)** Quantitative analysis of the xenografted tumor weight. **(D, E)** qRT-PCR analysis of miR-17-5p/4443 **(D)** and western blot analysis of TIMP2 expression **(E)**, left and middle) in each group and their quantification; Pearson’s correlation scatter plot of the fold changes of miR-17-5p, miR-4443 and TIMP2 protein tumor tissues. **(F)** HE staining and immunohistochemical analyses of Ki-67 and TIMP2 in primary tumors (n = 5) derived from the different groups. **(G, H)** Quantification of Ki-67 and TIMP2 intensity in figure. All data are represented as mean ± SD. *P < 0.05, ***P < 0.001.

We next investigated whether miR-17-5p and miR-4443 has an influence on tumor growth *in vivo*. TE-10 cells were pretreated with miR-17-5p lentivirus, miR-4443 lentivirus or control lentivirus. These pretreated cells were subcutaneously injected into the inguinal folds of the nude mice. The flowchart of the whole experiment was shown in **Figure 5A**. 28 days after the implantation, the implanted tumors were completely harvested and measured the weight and diameter. As shown in **Figures 5B, C**, miR-17-5p and miR-4443 overexpression group have a relatively high rate of tumor growth comparing to the control group. We then examined the effect of miR-17-5p and miR-4443 on TIMP2 expression and ESCC malignancy. QRT-PCR and Western blot shows that the overexpression of miR-17-5p and miR-4443 significantly down-regulated TIMP2 expression in xenografted tumor tissues (**Figures 5D, E**). These tumor tissues were then embedded in paraffin for H&E staining and immunohistochemical examination. H&E staining showed increased mitosis ratio in both miR-17-5p and miR-4443 overexpressing group compared to control group (**Figure 5F**). As shown in **Figures 5F-H**, higher level of miR-17-5p or miR-4443 resulted in decreased TIMP2 level and higher Ki-67 level. Taken together, these results further confirmed that miR-17-5p and miR-4443 acted as oncomiRs to regulate the progression of ESCC cells by targeting TIMP2.

## Discussion

Esophagus cancer is one of the most lethiferous malignant tumors all over the world, especially in East Asia like China. ESCC accounts for most of the EC patients. With the advancement of diagnostic techniques and the development of surgery as well as the application of molecular targeted drug and immunotherapy, the survival rate of ESCC patients has been greatly extended. However, the specific mechanism of the development of ESCC remains unknown. The quality of life of ESCC patients will seriously be degraded if tumor recurrence occurred. Current clinical treatment lacks effective therapy to inhibit metastasis. Our research provides a new potential way to inhibit ESCC metastasis.

Recent studies have shown the importance of miRNA in carcinogenesis and cancer development. For example, miR-148a might play its oncogenic role by targeting AVR1 in ESCC (16). miR-1224-5p inhibits tumor progression by targeting the TNS4/EGFR axis (17). There are also several types of research confirmed the oncogenetic roles of miR-17-5p. For example, in pancreatic cancer miR-17-5p enhance its proliferation by disrupting RBL2/E2F2-repressing complexes (13). And miR-17-5p can modulate NF-κB signaling in gastric cancer (18). Although there are few researches showed the opposite role of miR-17-5p in certain cancers (12, 19), it is reported that miR-17-5p can serve as prognostic indicators in ESCC (20). But the exact mechanism of miR-17-5p in ESCC remains unclear. miR-4443 is a rarely studied miRNA. It showed an oncogenetic role in breast cancer (21) and non-small cell lung cancer (10), and showed an opposite effect in ovarian cancer (22) and colon cancer (23). There is no research about miR-4443 in ESCC has been reported yet. In our study, we demonstrated that miR-17-5p and miR-4443 are stably up-regulated in ESCC tissues than in adjacent non-carcinoma tissues among all up-regulated miRNAs in the high-throughput miRNA sequencing. Both *in vitro* and *in vivo* experiments demonstrated the tumor-promoting effect of miR-17-5p and miR-4443 in ESCC. Because of the similar effect of miR-17-5p and miR-4443, we hypothesized that they may target the same protein. Then two independent bioinformatic tools were used to predict the potential target of the two miRNAs we studied. 11 genes were predicted to be targeted by both miR-17-5p and miR-4443. Among them, TIMP2, the inhibitor of matrix metalloproteinases (MMPs), was considered to be a potential target due to the known functions in cell proliferation and migration. Other target genes may also contribute to the effect of miR-17-5p and miR-4443 in ESCC cells. Among all results, TIMP2 was experimentally validated to be down-regulated by both of the miRNAs. Clinical ESCC tissues also showed lower expression of TIMP2 than adjacent non-carcinoma tissues. These results suggested that TIMP2 may serve as a tumor suppressor and be down-regulated during tumorigenesis, as has been shown by other researches (24–27). And targeting miR-17-5p and miR-4443 may be a potential therapy to control ESCC development. In the future, the mechanism of the up-regulation of miR-17-5p and miR-4443 in the ESCC patients need further studying.

TIMP2 (tissue inhibitor of metallopeptidase-2) is a member of the tissue inhibitor of metallopeptidases (TIMPs). The metastasis of cancer cells should invade into the extracellular matrix (ECM) firstly, and matrix metalloproteinases (MMPs) are essential and play core effect to degrade the ECM, paving a road for tumor cells to migrate into cycle system for distant metastasis (28). On the other hand, TIMPs, the inhibitor of MMPs, can reduce the degradation of ECM and therefore inhibit the invade of the primary tumor cells. There have been identified 4 members in the TIMP family (TIMP1-4) with different effects against different MMPs (29). TIMP2 has been reported to regulate the activity of MMP-2 (30), a significant factor to promote collagen degradation and lead to cancer cells’ dissemination (31). Researchers have found that MMP-2 is over-expressed in ESCC tumor tissues (32), and TIMP2 is down-regulated in both tissues and serum (33). Our research indicates that miR-17-5p and miR-4443 may be the reason and play a critical role to break the dynamic balance between TIMP2 and MMP-2 during ESCC development.

Taken together, our research demonstrated that miR-17-5p and miR-4443 are significantly upregulated in ESCC tissues, and serve as a tumor promoter by directly targeting TIMP2. Ectopic expression of miR-17-5p and miR-4443 may be one of the reasons for the up-regulation of MMP-2 in ESCC tissues. And the unbalanced state between TIMP2 and MMP-2 promote ESCC development and distant metastasis. Our research develops a new approach for understanding ESCC development and miR-17-5p and miR-4443 may serve as a potential target for ESCC therapy in future.

## Data Availability Statement

The original contributions presented in the study are included in the article/**Supplementary Material**, further inquiries can be directed to the corresponding authors.

## Ethics Statement

The studies involving human participants were reviewed and approved by Ethics Committee of the Jiangsu Cancer Hospital. The patients/participants provided their written informed consent to participate in this study. The animal study was reviewed and approved by Ethics Committee of the Jiangsu Cancer Hospital.

## Author Contributions

XC and LX planned the study. XW, JYH, and YL carried out the experiments. JWH and ML performed the data analysis and helped to draft the manuscript. XW wrote the manuscript. All authors contributed to the article and approved the submitted version.

## Conflict of Interest

The reviewers YW and YX declared a shared affiliation with author XC, to the handling editor at time of review.

The remaining authors declare that the research was conducted in the absence of any commercial or financial relationships that could be construed as a potential conflict of interest.

## Publisher’s Note

All claims expressed in this article are solely those of the authors and do not necessarily represent those of their affiliated organizations, or those of the publisher, the editors and the reviewers. Any product that may be evaluated in this article, or claim that may be made by its manufacturer, is not guaranteed or endorsed by the publisher.

## Supplementary Material

Supplementary Table 1 Raw microRNA array data.
